# Feeding and light cycle disruptions have distinct effects on energy balance and neuroendocrine regulators in goldfish

**DOI:** 10.1007/s10695-026-01685-1

**Published:** 2026-04-24

**Authors:** Nuria Saiz, Esther Isorna, Lisbeth Herrera-Castillo, María Jesús Delgado, Ignacio Ruiz-Jarabo, Nuria de Pedro

**Affiliations:** 1 Department of Genetics, Physiology and Microbiology, Faculty of Biological Sciences, Complutense University of Madrid, 28040 Madrid, Spain; 2 Department of Psychology, Faculty of Biomedical and Health Sciences, Universidad Europea de Madrid, Madrid, Spain; 3 Department of Marine Biology and Aquaculture, Institute of Marine Sciences of Andalusia—Spanish National Research Council (ICMAN-CSIC), 11519 Puerto Real, Spain

**Keywords:** Fish, Growth, Chronodisruption, Feeding regulation, Circadian system, Photoperiod

## Abstract

The synchronization of biological rhythms to environmental cues is essential for maintaining temporal homeostasis. However, the specific neuroendocrine and metabolic effects of circadian misalignment in fish remain poorly understood. This study investigated the effects of chronodisruption on energy homeostasis in goldfish (*Carassius auratus*). Fish were exposed for 53 days to three conditions: Control (12L:12D photoperiod, feeding at ZT 1); Random Feeding (RF; 12L:12D, feeding at random times) and continuous light (LL, feeding at ZT 1). Feed intake, metabolic rate, and locomotor activity were monitored as indicators of energy input and expenditure. Biometric indices (weight and length gain, growth, nutritional and hepatosomatic indices) were calculated. The expression of feeding and growth regulating genes was also analyzed in hypothalamus (*hcrt, npy, crh, pomca, cartpt1,* and *cartpt2*), telencephalon (*cnr1*), liver (*igf1*, and *lepa1*), and intestine (*cck1* and *ghrl*). Plasma and liver metabolites (triglycerides, glycogen, lactate, and glucose) and plasma cortisol levels were analyzed to assess metabolic and stress responses. Both RF and LL groups showed elevated metabolic rate and feed intake, accompanied by changes in the expression of feeding regulators that favored orexigenic signals. However, only RF fish showed increased growth, weight, hepatosomatic index, and plasma glucose, suggesting a positive energy balance. LL fish showed growth similar to controls, likely due to a greater increase in energy expenditure. Overall, our results show that disruption of temporal feeding or light cues alters energy balance, with different physiological outcomes depending on the type of disruption. This study highlights the critical role of circadian alignment in maintaining energy homeostasis in goldfish.

**Supplementary Information:**

The online version contains supplementary material available at 10.1007/s10695-026-01685-1.

## Introduction

One of the main factors determining efficiency and cost in the context of aquaculture is feeding management. Optimal feed consumption and utilization are key to fish growth, maximizing production in fish farms. As in other vertebrates, feeding in fish is regulated by homeostatic mechanisms, primarily the neuroendocrine circuits involving orexigenic and anorexigenic factors (Delgado et al. 2017; Soengas et al. 2024; Volkoff 2024). Some of these neuroendocrine signals are produced in the brain, particularly in the hypothalamus. These include the orexigenic neuropeptide Y (NPY) and orexins (hypocretins); as well as the anorexigenic proopiomelanocortin (POMC), cocaine-and amphetamine-regulated transcript (CART), and corticotropin-releasing hormone (CRH). At the peripheral level, gastrointestinal signals like cholecystokinin (CCK) and hepatic signals such as leptin play a significant role in the regulation of feeding.

In addition, food intake is modulated by the circadian system, enabling animals to anticipate the arrival and availability of nutrients and thereby optimize their utilization (Delgado et al. 2017; Challet 2019). A close interplay exists between homeostatic and circadian processes, and consequently, the circadian system is a key regulator of metabolic health and energy balance (Marcheva et al. 2013). Evolutionarily, the circadian system developed in response to cyclic environmental changes caused by the Earth’s rotation, allowing animals to adapt their physiology, metabolism, and behavior accordingly (Sánchez-Vázquez et al. 2019). It consists of a complex network of endogenous oscillators distributed across central and peripheral tissues that generate circadian rhythms with a period of nearly 24 h (Mendoza 2006; Isorna et al. 2017), controlling the timing of various physiological functions. At the core of these oscillators are molecular clocks, driven by transcriptional-translational feedback loops of "clock genes", which last approximately 24 h (Buhr and Takahashi 2013; Isorna et al. 2017). Importantly, endogenous clock gene rhythms can persist for several days under constant conditions (i.e., free-running conditions; Buhr and Takahashi 2013).

These biological oscillators must be “set on time” by different environmental cues, known as *Zeitgebers* or synchronizers, which determine the phase and period of biological rhythms. The main *Zeitgeber* is the light–dark cycle, which entrains the so-called light-entrainable oscillators (LEOs). In mammals, the main LEO is a master oscillator located in the suprachiasmatic nucleus of the hypothalamus, which coordinates the rest of the endogenous clocks. However, in non-mammalian vertebrates such as teleosts, the system seems to be more decentralized, consisting of a network of non-hierarchically organized oscillators widely distributed (Toloza-Villalobos et al. 2015; Isorna et al. 2017). Nonetheless, in fish, the light/dark cycle is also a key synchronizer, as most neural oscillators behave as LEOs (Vera et al. 2013; Frøland Steindal and Whitmore 2019; Saiz et al. 2021).

Food sources in both nature and fish farming can vary in time in a predictable rhythmic manner, requiring animals to anticipate food availability to optimize their chances of survival (Stephan 2002). This way, the feeding schedule is an important synchronizer for fish and other vertebrates, particularly for the peripheral clocks, which are primarily food-entrainable oscillators (FEOs; Damiola et al. 2000; Gómez-Boronat et al. 2018, 2022). Recent evidence highlights the significance of these peripheral oscillators, which, when entrained by food, can anticipate the time of feeding to optimize foraging, digestion, metabolism, and nutrient utilization (Chaix et al. 2019). This anticipation is often observed as an increase in locomotor activity a few hours before feeding time, known as food anticipatory activity (FAA; Sánchez-Vázquez et al. 1997; Mendoza 2006). The FAA can persist in the absence of a light/dark cycle, and it is maintained even under prolonged fasting conditions. This indicates that it is not triggered by external signals or induced by the intake itself, but rather, it is generated by the endogenous timekeeping system (Aranda et al. 2001). In addition to FAA, other pre-mealtime adaptations also occur, including changes in metabolic parameters, glucocorticoid release (Mendoza 2006), increased secretion of some neuroendocrine factors, and numerous digestive events (Patton and Mistlberger 2013; Navarro-Guillén and Yúfera 2025), all to prepare for the upcoming meal.

The concept of chronodisruption refers to the disturbance of the circadian system resulting from mismatches between endogenous circadian rhythms and environmental cycles. This phenomenon has been extensively studied, mainly in mammals, and has been shown to impair numerous physiological, neuroendocrine, metabolic, and behavioral processes, given the large range of functions that depend on circadian rhythms (Albrecht 2012). Many animals organize their activity and rest patterns around the light/dark cycle, and most physiological functions are synchronized to these rhythmic cycles. Thus, one of the main types of chronodisruption involves disturbances of the natural photoperiod. This disruption is particularly evident in mammals, where the effects of artificial light at night (ALAN) have been extensively reported (West et al. 2017; Kelly et al. 2018). ALAN can blunt the regular rhythms of glucocorticoids, melatonin, and locomotor activity. This disruption results in major metabolic dysregulations leading to obesity, insulin resistance, chronic inflammation, or increased stress (Fonken and Nelson 2014). The clock genes may be at the core of such effects, as genetic alterations in circadian clocks have been shown to cause adverse metabolic outcomes in mammals, such as increased weight gain, metabolic syndrome, modifications in adipose deposits, and in glycaemia, in mammals (Kolbe and Oster 2019). In teleost fish, ALAN also affects growth, induces changes in the immune system, leads to retinal degradation and increased formation of tumors, among others (Khan et al. 2018; Sánchez-Vázquez et al. 2019), presenting a threat to aquatic ecosystems (Khan et al. 2018; Sánchez-Vázquez et al. 2019; Zapata et al. 2019; Stanton and Cowart 2024). Chronodisruption due to abnormal lighting schedules is not only present in the ecosystems, but also in some aquaculture practices, particularly during the larval stage. For example, European sea bass larvae are often reared under constant darkness, while gilthead sea bream larvae are sometimes kept under constant light during the first days after hatching. These artificial lighting conditions can affect the expression of clock genes and the timing of feeding behavior (Morretti et al. 1999; Mata-Sotres et al. 2015). Another form of chronodisruption arises from the lack of a regular 24 h fasting/feeding cycle. Mistimed meals, specifically meals consumed during the natural resting period (i.e. the daytime for nocturnal animals and the nighttime for diurnals), seriously affect the energy balance of organisms (Morton et al. 2014). The effects of this feeding chronodisruption on fish remain unclear, but some studies have demonstrated that meals provided during the night or at random times disturb the daily rhythms of clock genes (Gómez-Boronat et al. 2018; Saiz et al. 2021).

The goldfish (*Carassius auratus*) is a cyprinid species widely employed in research and of special interest in aquaculture as an ornamental species (Blanco et al. 2018; FAO 2023, 2024), that has primarily diurnal habits (Iigo and Tabata 1996). The effects of circadian misalignment under chronodisruptive conditions have been extensively studied in this species, affecting the rhythms of locomotor activity, multiple hormones, and clock genes in central and peripheral oscillators (Saphiro and Hoffman 1975; Delahunty et al. 1978; Vera et al. 2007; Velarde et al. 2010; Nisembaum et al. 2012; Tinoco et al. 2014; Sánchez-Bretaño et al. 2015a, 2015b; Gómez-Boronat et al. 2018, 2022; Saiz et al. 2021, 2023; Alonso-Gómez et al. 2022). Alterations in anxiety-like behavior and stress axis associated with circadian disruption have also been reported (Saiz et al. 2021; 2023). However, the effects of circadian misalignment on energy homeostasis remain unclear. Thus, the aim of this study was to investigate the effects of chronic chronodisruption on energy balance in goldfish. For this purpose, two protocols inducing circadian misalignment were used, one removing the light/dark cycle (continuous light) and the other removing the feeding/fasting schedule (random feeding). We evaluated the impact of these conditions on weight gain and growth, considering the balance between energy intake (feed consumption) and energy expenditure (metabolic rate and locomotor activity). To explore the mechanisms underlying changes in feed intake caused by chronodisruption, we analyzed the expression of orexigenic and anorexigenic genes. We also assessed metabolic consequences by measuring carbohydrate and lipidic metabolites in plasma and liver. Finally, plasma cortisol levels were determined to evaluate whether chronodisruption acted as a physiological stressor in goldfish.

## Material and methods

### Animals and housing

Goldfish (4.4 ± 0.1 g body weight, bw) were procured from ICA S.A. (Madrid), and were housed in 60 L fish tanks (8–10 individuals/tank), with filtered and aerated water maintained at 21 ± 1 °C, and under a controlled photoperiod (12 h of light and 12 h of darkness, 12L:12D, lights on at 8:00 h). Fish were fed once daily at *Zeitgeber* Time 1 h (ZT 1, i.e. 1 h after lights-on) by automatic feeders with commercial granulated feed (1.5% bw, Sera Pond Biogranulat, Heisenberg, Germany), with the ration adjusted weekly according to the total biomass of fish in each tank. All fish were identified individually using a subcutaneous injection of black ink (Eternal Ink, Brighton, USA) at different locations. All fish were acclimated to these conditions for 20 days before the start of the experiment. The procedures complied with the Guidelines of the European Union Council (2010/63/EU) and the Spanish Government legislation (RD53/2013) on the use of animals in scientific proposals and were approved by the Animal Experimentation Committee of the Complutense University and the Community of Madrid (PROEX 317.7/23).

### Experimental design

To investigate the effect of chronodisruption on energy homeostasis, goldfish were divided into 3 experimental groups (*n* = 20, 2 tanks per group) under different lighting conditions and feeding schedule during 53 days: (1) Control group: maintained under the same acclimation conditions (12L:12D photoperiod and fed (1.5% bw) once daily at ZT 1; (2) Random Feeding (RF) group, maintained under 12L:12D photoperiod and fed (1.5% bw) once daily at a random times (generated by the RAND function of Microsoft Excel®), with approximately half of the feeding occurring during the night; and (3) Continuous light (LL) group, exposed to 24 h of light (24L) and fed (1.5% bw) daily at the same time that the control group, i.e. circadian time (CT) 1 h. As this group was under constant illumination, a subjective circadian time scale was applied, with CT 0 being the last time the lights were turned on.

Locomotor activity of the animals was recorded for 9 days before the start of the experiment and the first 35 days of chronodisruption conditions. Feed intake was quantified on day 32, and metabolic rate on days 40–41. At the end of the experiment (day 53), animals were anaesthetized by immersion (MS-222, 0.16 g/L, Sigma-Aldrich, Madrid, Spain), weighed and measured at ZT 5 (or CT 5), and blood was collected for plasma cortisol and metabolite analysis. At the end of the experiment (day 53), animals were not fed on the day of sampling, resulting in a 29 h fasting period under all experimental conditions to avoid potential acute effects of feeding. Subsequently, the animals were euthanized by anesthetic overdose (MS-222, 0.4 g/L) to obtain hypothalamus, telencephalon, liver, and anterior intestine for expression analysis of feeding regulators. The liver was also weighed and sampled for metabolite analysis. Tissue samples were frozen in liquid nitrogen and preserved at −80 °C until analysis.

### Locomotor activity

The locomotor activity of the fish was recorded directly in the maintenance tanks, where fish were continuously housed from their arrival at the laboratory, through both the acclimation and experimental periods, as previously described (Saiz et al. 2022). Photocells (Omron, E3S-AD12, Kyoto, Japan) were fixed to the aquaria walls. Two photocells were placed in the upper part of each tank to register the locomotor activity associated with the feeder, while the remaining four were placed at 3–9 cm above the bottom of each tank to record the general locomotor activity. These photocells emitted a continuous beam of infrared light and transmitted a signal whenever the beam was interrupted. The resulting pulse was registered by an actimeter and a data acquisition software (Adq16, Micronec, Madrid, Spain), for further analysis. To prevent any visual interferences, all the aquaria were covered with opaque paper.

### Feed intake

To evaluate feed intake, the method described in Saiz et al. (2022) was employed. Briefly, 24-h fasted goldfish were isolated in 5-L buckets at ZT 1 (or CT 1 for the LL group) and allowed to acclimate for one hour. A pre-weighed excess of food (3% bw) was then provided and after 2 h, the remaining food was retrieved. Then another 3% bw was added, and 6 h later, the remaining food was collected again. All unconsumed food was left to dry for 24 h at 70 °C and weighed. Feed intake during the 0–2, 2–8, and 0–8 intervals, was calculated using the formula: ${\boldsymbol{F}}{\boldsymbol{I}}=({\boldsymbol{W}}{\boldsymbol{i}}-{\boldsymbol{W}}{\boldsymbol{f}})\times {\boldsymbol{f}}$, where ***FI*** represents feed intake, ${\boldsymbol{W}}{\boldsymbol{i}}$ represents the initial dry weight of food, ${\boldsymbol{W}}{\boldsymbol{f}}$ represents the final dry weight of food, and ${\boldsymbol{f}}$ represents the specific dilution factor of the food. The dilution factor was determined by adding a known amount of food to 5-L buckets without fish and collecting the remains after 2 and 6 h.

### Biometric parameters

To assess fish growth and physiological condition, we calculated several biometric parameters. Gain of body weight and standard length were calculated as a percentage relative to the initial values (day 3), chosen to allow for recovery from potential acute stress following transfer to experimental conditions. The specific growth rate was determined by the formula ${\boldsymbol{S}}{\boldsymbol{G}}{\boldsymbol{R}}=[\mathbf{l}\mathbf{n}({\boldsymbol{W}}{\boldsymbol{i}}-{\boldsymbol{W}}{\boldsymbol{f}})/{\boldsymbol{t}}]\times 100,$ where ${\boldsymbol{W}}{\boldsymbol{i}}$ and ${\boldsymbol{W}}{\boldsymbol{f}}$ are initial and final body weight and ${\boldsymbol{t}}$ = 50 days. The nutritional index (condition factor) was determined by${\boldsymbol{N}}{\boldsymbol{I}}=({\boldsymbol{W}}{\boldsymbol{f}}\times 100)/({\boldsymbol{L}}{\boldsymbol{f}}^3)$, where ${\boldsymbol{L}}{\boldsymbol{f}}$ is the final standard length of fish. The hepatosomatic index (HSI) was calculated as the percentage of the total body weight of the fish that corresponded to the liver.

### Metabolic rate

Oxygen consumption (MO_2_) on 24-h fasted goldfish (n = 8/group) was measured using an intermittent-flow respirometry system (Loligo Systems, Viborg, Denmark), previously validated for this species in our laboratory (Herrera-Castillo et al. 2024). The system consisted of four methacrylate chambers in which fish were introduced individually, and four optical sensors that measured oxygen concentration inside each chamber. The temperature was maintained constant at 21 ± 1 °C during the assay. The system was placed inside a 70 L tank filled with oxygen-saturated water. For the information acquisition of the oxygen and temperature sensors, the Witrox-4 module and AutoResp™ software were used.

### Plasma and liver metabolites

Plasma levels of glucose, triglycerides and lactate were determined by colorimetric commercial kits adapted to a microplate format (1,001,330, 1,001,310, MD41011, Spinreact, Girona, Spain), as previously reported (Saiz et al. 2022). Liver glycogen and triglycerides concentrations were determined as previously explained (Saiz et al. 2022). In short, livers were homogenized by sonication in 7.5 vols of ice-cooled 0.6 N perchloric acid and the pH was neutralized with 7.5 vols of ice-cooled 1 N potassium bicarbonate. The homogenate was centrifuged at 13,500 g and 4 °C for 4 min, and the resulting supernatant was stored for further analysis. Liver triglycerides were measured using a commercial kit (1001310, Spinreact). To assess liver glycogen levels, we employed the Keppler and Decker method (Keppler and Decker 1974), which involves measuring the glucose obtained after glycogen breakdown using amiloglucosidase (Sigma-Aldrich), and then subtracting the free glucose levels. Tissue glucose concentration was measured using a commercial kit (MD41011, Spinreact).

### Plasma cortisol

Plasma cortisol was measured by duplicate according to the method described in Ruiz-Jarabo et al. (2022). In summary, 3 N HCl was added at a 1:1 ratio to the samples and allowed to stand for 10 min at room temperature. Next, a 10 × volume of methanol was added to each sample and centrifuged twice at 12,000 rpm and 4 °C for 10 min. The supernatants were collected and used for ELISA (Cortisol ELISA Kit, Cayman Chemical, Michigan, US), following the commercial protocol. The free cortisol values were within the range described by the manufacturer (10–800 ng/mL).

### Gene expression analysis

The relative mRNA abundance of *hcrt* (orexin gene)*, npy, crh, pomca, cartpt1 and cartpt2* (CART genes) in hypothalamus, *cnr1* in telencephalon, *igf1* and *lepa1* in liver and *cck1* and *ghrl* in anterior intestine were analyzed by qPCR, as previously described (Saiz et al. 2022). Total RNA from telencephalon, hypothalamus, anterior intestine, and liver was isolated using TRI® Reagent (Sigma-Aldrich) and then treated with RQ1 RNase-Free DNase (Promega, Madison, USA). Reverse transcription of RNA was carried out using random primers (Invitrogen, Waltham, USA), RNase inhibitor (Promega), and SuperScript IV Reverse Transcriptase (Invitrogen) starting with 0.3 µg of total RNA into a 25 μL final volume. Real-time quantitative PCRs were performed duplicate on each sample using iTaqTM Universal SYBR Green Supermix (Bio-Rad, Hercules, USA), into a 96-well plastic plate, in a CFX96TM Real-Time qPCR System (Bio-Rad). The reaction mix included 1 µL of cDNA and forward and reverse primers 0.5 µM (Sigma-Aldrich), to a final volume of 10 µL. Each PCR plate contained a standard dilution curve to verify the efficiency of PCR reactions (90–105%) and negative controls such as water and pre-RT RNA. The RT-qPCR protocol included an initial denaturation step of 95 °C for 30 s, followed by 40 cycles of a two-step amplification program (95 °C for 5 s and 60 °C for 30 s). To ensure specificity, melting curves were monitored systematically (temperature gradient at 0.5 °C/5 s from 70 to 90 °C). The Gene Data Bank reference numbers and primer sequences for target and housekeeping genes are presented in Table 1. The 2^−∆∆Ct^ method (Livak and Schmittgen 2001) was used to calculate relative mRNA expression, where all data were normalized to the mean of the control group.

**Table 1:** Primers used in the RT-qPCR for housekeeping and target genes

Gene name	Gene symbol	Access number (GenBank)	Sequence (5′➔3’)		Product (bp)	Efficiency
*actin beta*	*actb*	AB039726.2	F	CAGGGAGTGATGGTTGGCA	168	104.7%
			R	AACACGCAGCTCGTTGTAGA		
*eukaryotic translation elongation factor 1 alpha*	*eef1a*	AJ431209	F	CCCTGGCCACAGAGATTTCA	101	97.2%
			R	CAGCCTCGAACTCACCAACA		
*cocaine and amphetamine regulated transcript 1*	*cartpt1*	AY033816	F	GTGCCGAGATGGACTTTGAC	97	103.4%
			R	AGCTGCTTCTCGTTGGTCAG		
*cocaine and amphetamine regulated transcript 2*	*cartpt2*	AF098629	F	GGCTCTGCTCGTTGCCTTT	121	105.7%
			R	CGGTTTGCTCCAGCTCAGA		
*cannabinoid receptor 1*	*cnr1*	AY674057.1	F	GCAGCGTCATCTTCGTCTAC	90	94.3%
			R	CGCCTCCTAACTTGAACA		
*cholecystokinin 1*	*cck1*	U70865	F	GAGGATGATGAAGAGCCCCG	112	106.1%
			R	TGTTGCCCATGGACTTGCTT		
*corticotropin-releasing hormone*	*crh*	AF098629	F	GGCTCTGCTCGTTGCCTTT	121	94.1%
			R	CCCTAAGCGTGCCAAAACC		
*ghrelin*	*ghrl*	AF454389	F	TTCATGATGAGTGCTCCGTTC	124	104.7%
			R	GTCAGAATTCAAGTGGCGAATC		
*hypocretin*	*hcrt*	DQ923590.1	F	ACTGCACAGCCAAGAGAGTTCA	166	105.8%
			R	GTTATTAAAGCGGCCGATATGC		
*insulin-like growth factor 1*	*igf1*	AF001006.1	F	CAGGGGCATTGGTGTGA	153	103.4%
			R	GCAGCGTGTCTACAAGC		
*leptin a1*	*lepa1*	FJ534535.1	F	AGCTCCTCATAGGGGATC	192	105.1%
			R	TAGATGTCGTTCTTTCCTTA		
*neuropeptide Y*	*npy*	M87297	F	TTCGTCTGCTTGGGAACTCT	151	97.4%
			R	TGGACCTTTTGCCATACCTC		
*proopiomelanocortin a*	*pomca*	AJ431209	F	CTCACCACTGACGAGAACATCTTG	121	96.2%
			R	CGGTTTGCTCCAGCTCAGA		

*F* Forward, *R* Reverse

### Data analysis

The number of individuals per group and replicate tanks was determined based on previous studies using similar experimental designs in goldfish (Gómez-Boronat et al. 2019; Saiz et al. 2022; Herrera-Castillo et al. 2025b) and informed by power analysis. Tank homogeneity within each experimental group was verified using Student's t-test or Mann–Whitney U test, confirming the absence of significant differences between replicate tanks and justifying the use of individual fish as experimental units. The data are presented as mean ± standard error of the mean (SEM). The normality and homoscedasticity of data were confirmed by the Shapiro–Wilk and Levene tests, and data were adjusted to a logarithmic or square root scale if needed. To compare the data series of chronodisrupted groups (LL and RF) with the control, Student’s t-test was employed for parametric data, while the Mann Whitney-U test was applied for non-parametric data. These pairwise comparisons were pre-planned and address independent biological hypotheses. No statistical comparison between RF and LL groups was performed. The statistical tests and transformations stated, as well as the graphical representation of the data, were performed using SigmaPlot© 12 software. Locomotor activity data were analyzed using El Temps® software, which obtains profiles of average daily activity rhythms, actograms, and periodograms. General locomotor activity actograms were generated using the combined activity detected by the four photocells at the bottom of the tank, while feeding-associated actograms were generated using the combined activity detected by the two photocells near the feeder. Chi-square periodograms were used to examine the existence of daily rhythms in activity with a significance threshold of *p* < 0.05.

## Results

**Fig. 1:**
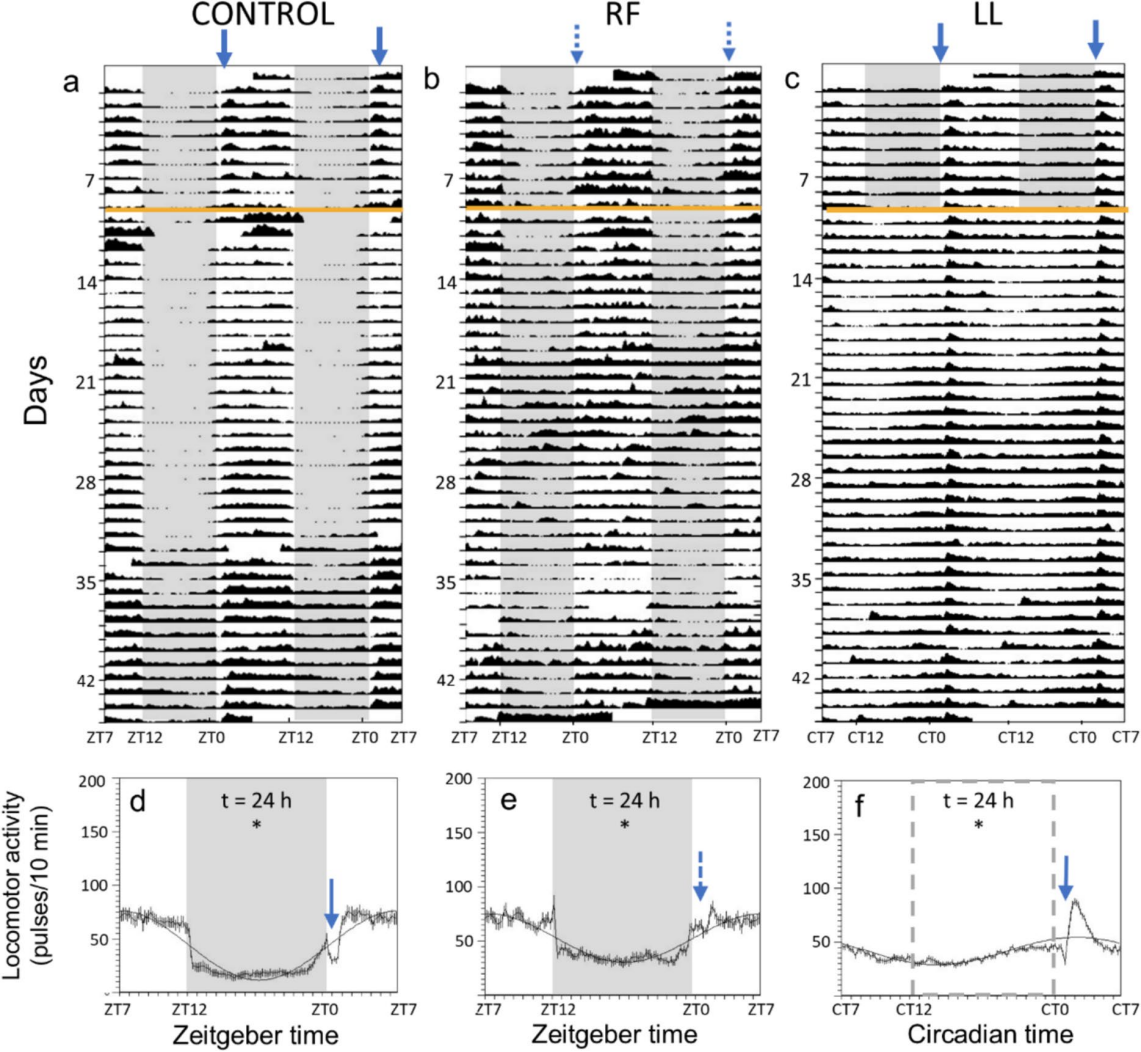
General locomotor activity of goldfish under three lighting and feeding regimes: (CONTROL, RF-random feeding, and LL, continuous light). **(a-c)** Representative actograms (double representation, 48 h time scale). The orange line represents the start of the experimental conditions. **(d-f)** Average profile of general locomotor activity during the experimental period, represented by the mean ± SEM (*n* = 35 days per group), bold black line indicates a periodic sinusoidal function wave. The shaded area indicates scotophase (ZT 12—ZT 0) and the blue arrows indicate mealtime (ZT 1). The shaded area in LL (c and f) and the arrow in RF (b and e) are shown in dashed line, as these groups lack scotophase and fixed feeding time, respectively. t is the period of the rhythm (in hours) when it is significant according to Sokolove-Bushell periodograms (* *p* < 0.05)

**Fig. 2:**
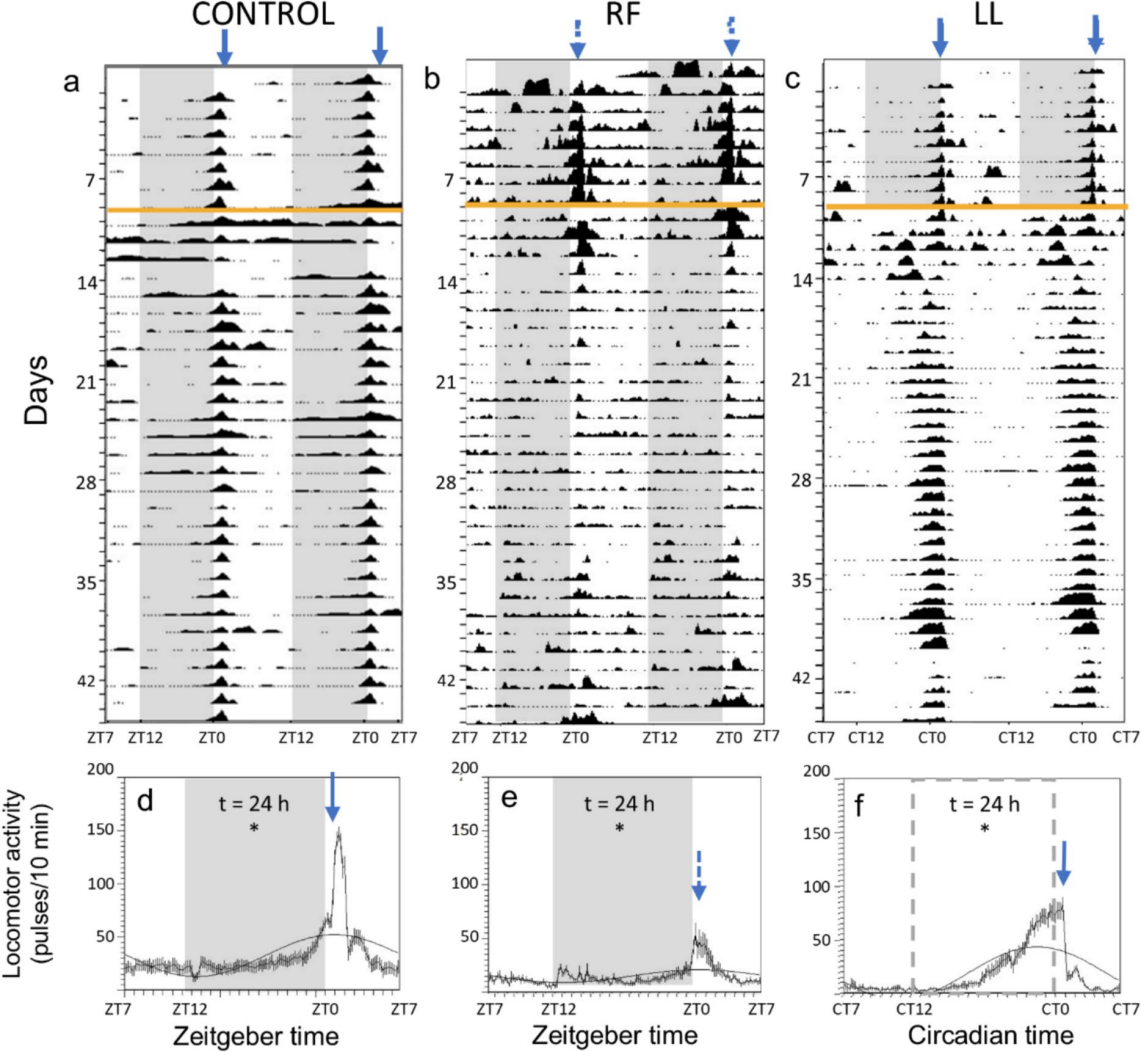
Feeding-associated locomotor activity of goldfish under three lighting and feeding regimes: (CONTROL, RF-random feeding, and LL, continuous light). **(a-c)** Representative actograms (double representation, 48 h time scale). The orange line represents the start of the experimental conditions. **(d-f)** Average profile of feeding-associated locomotor activity during the experimental period, represented by the mean ± SEM (*n* = 35 days per group), bold black line indicates periodic sinusoidal function wave. The shaded area indicates scotophase (ZT 12—ZT 0) and the blue arrows indicate mealtime (ZT 1). The shaded area in LL (c and f) and the arrow in RF (b and e) are shown in dashed line, as these groups lack scotophase and scheduled feeding time, respectively. t is the period of the rhythm (in hours) when it is significant according to Sokolove-Bushell periodograms (* *p* < 0.05)

Figure 1 illustrates the general locomotor activity of each experimental group during 9 days before the start of the experiment and the 35 first days of chronodisruption conditions. The activity pattern of the three groups (control, LL and RF) during 9 days before the experimental conditions is clearly diurnal (Fig. 1a-c), with higher activity during the light phase (ZT 0–12) and lower during the dark phase (ZT 12–0. During the experimental period, all three groups showed significant 24-h rhythms of locomotor activity (Fig. 1d-f). The RF group showed a similar daily profile to the control group, but attenuated (Fig. 1b,e), while the LL group exhibited constant levels of activity throughout the 24 h, except for a peak in general activity after feeding time (Fig. 1c,f). The locomotor activity near the feeders is shown in Fig. 2. In the 9 days leading up to the experiment, all three groups displayed a marked increase in activity a few hours before food delivery, peaking just before ZT 1, indicating FAA (Fig. 2a-c, 9 first days). After switching to continuous light, the LL group initially had disorganized feeder-associated activity, but FAA reappeared around 4 days into the LL conditions, becoming more intense and starting earlier than in control group (Fig. 2c). In contrast, the RF group maintained a peak of activity before ZT 1 for about 5 days (days 9–14) after the start of random feeding, after which it progressively attenuated until disappearing (Fig. 2b). When activity profiles were averaged across the whole experimental period, all three groups exhibited significant 24-h rhythms in their feeding-associated locomotor activity profiles (Fig. 2d-f). However, in the RF group this was driven by the persistence of feeding-anticipatory activity from the previous ZT 1 schedule during the first days, whereas feeder-associated activity was not rhythmic during the later phase of the experiment (Fig. 2e).

**Fig. 3:**
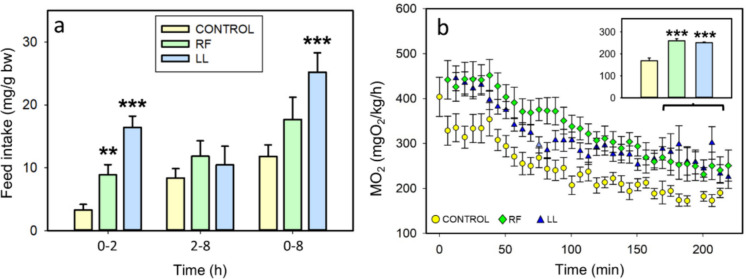
Effect of randomly-timed meals (RF) and continuous light (LL) on feed intake and metabolic rate in goldfish. (**a**) Feed intake relative to body weight (bw) in the 0–2 h, 2–8 h, and 0–8 h intervals (mean + SEM n = 10–15/group). (**b**) Metabolic rate during 250 min. Data points represent mean + SEM (n = 14–15/group), and the bar graphs indicate the mean + SEM of the data from 180–250 min interval. ** *p* < 0.01, *** *p* < 0.001 (Student’s t-test, compared to control group)

Both the random feeding (RF) and continuous light (LL) groups showed significantly higher feed intake during the first 2 h of the assay (*p* < 0.01 and *p* < 0.001, respectively), with LL fish exhibiting an approximately fivefold increase compared to control fish (Fig. 3a). No significant modifications in feeding were observed during the discrete interval 2–8 h. Cumulative feed intake at 8 h was significantly higher in LL group compared to controls (*p* < 0.001). RF and LL fish exhibited a significantly higher MO_2_ compared to the control group throughout the 250-min analysis period. This increase was already evident during the first 30 min of measurements, suggesting an enhanced acute response to the testing conditions, (*p* < 0.001, Fig. 3b). MO_2_ progressively decreased as all fish acclimated to the chamber, as expected; and the elevated oxygen consumption in the RF and LL groups remained significantly higher than in controls during the final 70 min of recording (*p* < 0.01).

**Fig. 4:**
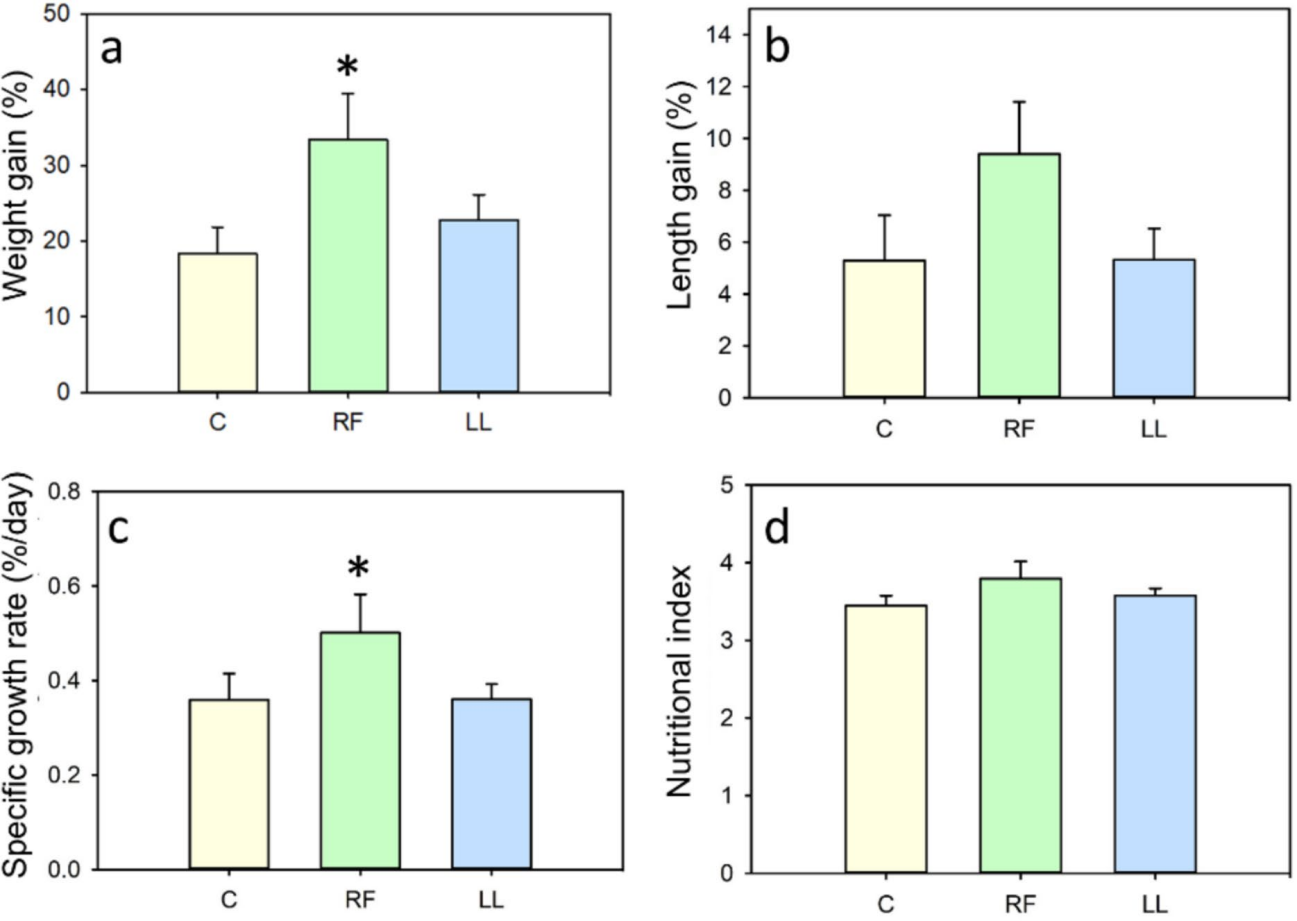
Effect of randomly-timed meals (RF) and continuous light (LL) on biometric parameters of goldfish. (**a**) Weight gain. (**b**) Length gain. (**c**) Specific growth rate (SGR). (**d**) Nutritional index. Data are expressed as mean + SEM. (*n* = 11–18/group) * *p* < 0.05 compared to control group, C (Student’s t-test used for a, c, d; Mann–Whitney U test used for b)

Regarding biometric parameters, randomly fed fish showed an increased weight gain (83%) and specific growth rate (39%) compared to the control group (Fig. 4a,c). No statistically significant differences were detected in length gain (Fig. 4b). No differences in any of these biometric parameters were found in fish exposed to continuous light (LL) when compared to the control group (Fig. 4a-d). The nutritional index was similar in the three groups (Fig. 4d).

**Fig. 5:**
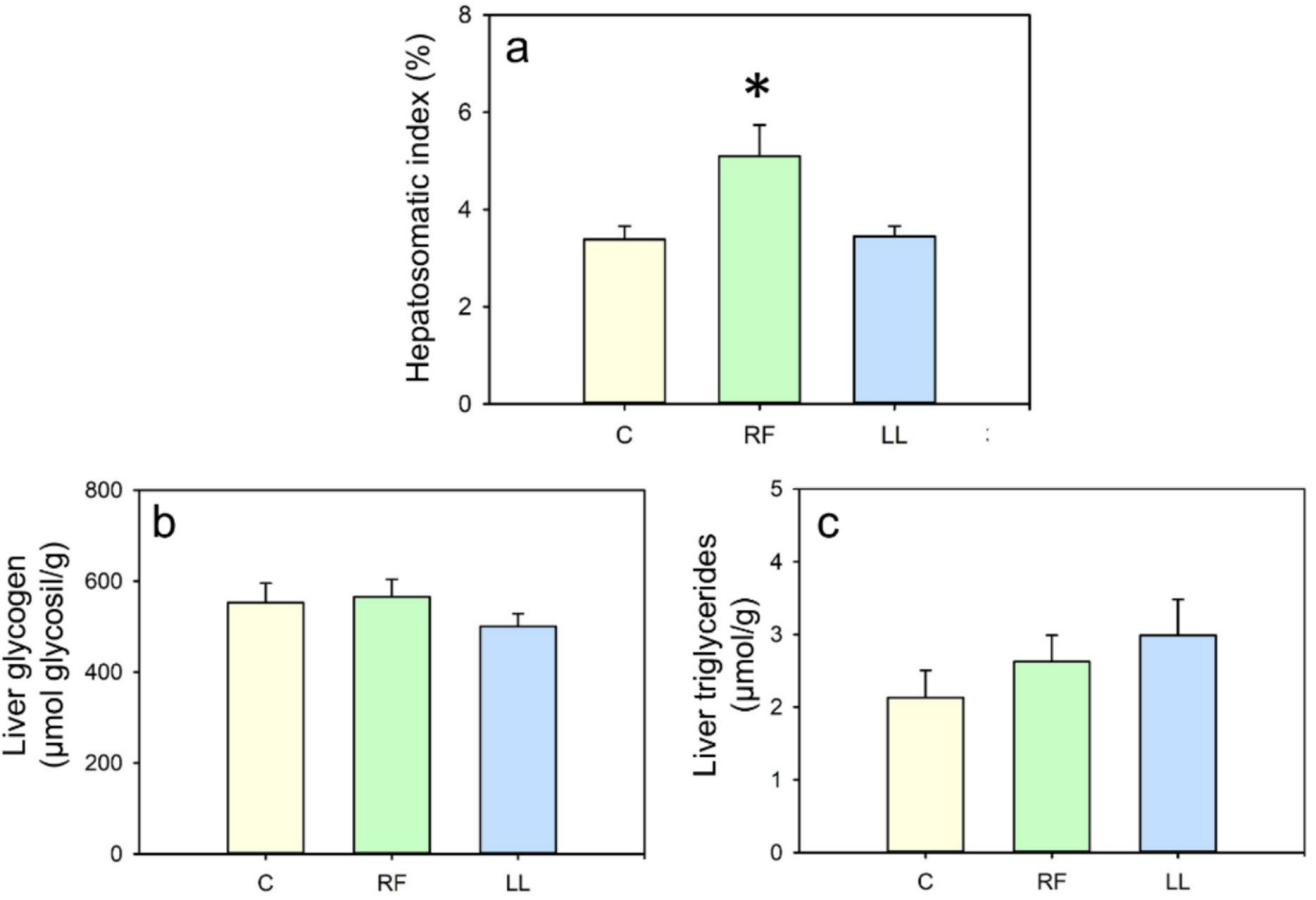
Effect of randomly-timed meals (RF) and continuous light (LL) in goldfish liver. (**a**) Hepatosomatic index. (**b**) Liver glycogen. (**c**) Liver triglycerides. Data are expressed as mean + SEM (*n* = 6–10/group). * *p* < 0.05 (Student’s t-test for all comparisons except for C vs. LL in panel a, where Mann–Whitney U was used)

Regarding liver parameters, RF fish showed an increased hepatosomatic index (51%, Fig. 5a). Despite this larger liver relative to the body weight, the hepatic concentration of glycogen and triglycerides in RF fish was not significantly different from that of the control group (Fig. 5b-c). However, due to the increased liver size, the total amount of glycogen and triglycerides stored in the liver of RF fish was likely higher than in controls. Conversely, the LL group did not show any significant differences in these parameters compared to the control group (Fig. 5a-c).

Table 2 shows plasma cortisol and metabolites in fish under randomly–timed meals and continuous light compared to controls. No statistically significant differences in cortisol levels were detected between groups; however, *p*-values close to the conventional significance threshold indicate numerically higher average cortisol levels in chronodisrupted fish compared with controls (*p* = 0.1 for RF and *p* = 0.08 for LL). Additionally, plasma glucose was significantly increased in the RF group (*p* < 0.05). Furthermore, both RF and LL groups showed significantly lower plasma triglyceride levels than the control group. Plasma lactate levels were similar in the three groups.

**Table 2:** Effect of randomly-timed meals (RF) and continuous light (LL) on plasma cortisol and metabolites in goldfish

	CONTROL	RF	LL
Cortisol (ng/ml)	61.21 ± 9.03	73.35 ± 7.86	79.24 ± 8.79
Glucose (mM)	2.09 ± 0.13	**2.52** ± 0.15 *	1.89 ± 0.6
Triglyceride (mM)	4.45 ± 0.58	**4.04** ± 0.25 *	**3.03** ± 0.45 *
Lactate (mM)	1.24 ± 0.13	1.36 ± 0.12	1.22 ± 0.12

Data are expressed as mean ± SEM. *n* = 6–9/group for metabolites, and 14–19 for cortisol analysis). * *p* < 0.05 (Student’s t-test was used for all comparisons except for glucose and the C vs. LL comparison of lactate, where Mann Whittney U was used)

**Fig. 6:**
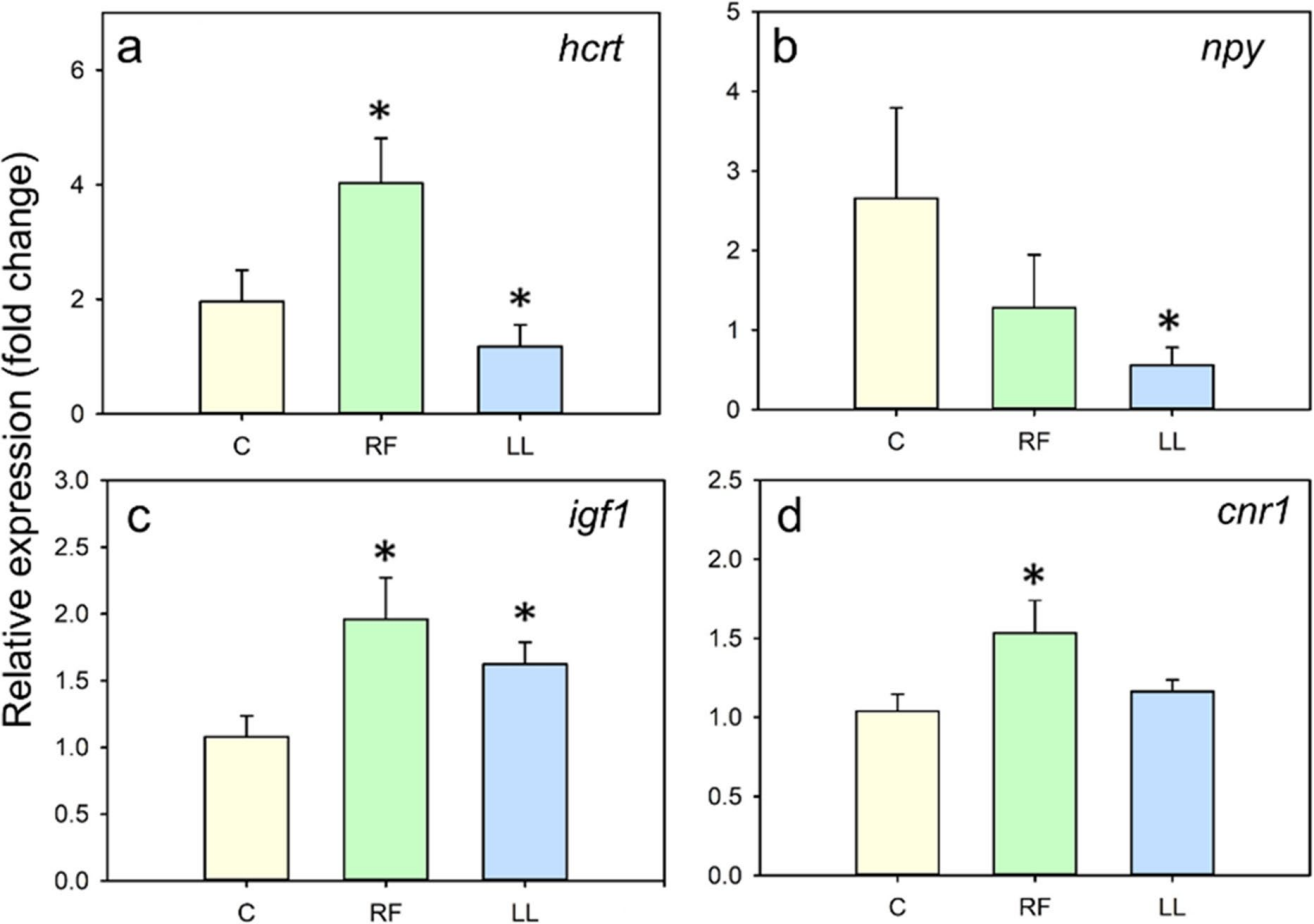
Effect of continuous randomly-timed meals (RF) and continuous light (LL) in expression of orexigenic and growth-related factors in goldfish. Relative mRNA abundance of (**a**) *hcrt* and (**b**) *npy* in hypothalamus, (**c**) *igf1* in liver, and (**d**) *cnr1* in telencephalon of goldfish. Data are expressed as mean + SEM. * *p* < 0.05 compared to control group, C (Student’s t-test used for all comparisons, except for b and for C vs. RF comparison in c, where Mann–Whitney U was used. *n* = 6–10/group

**Fig. 7:**
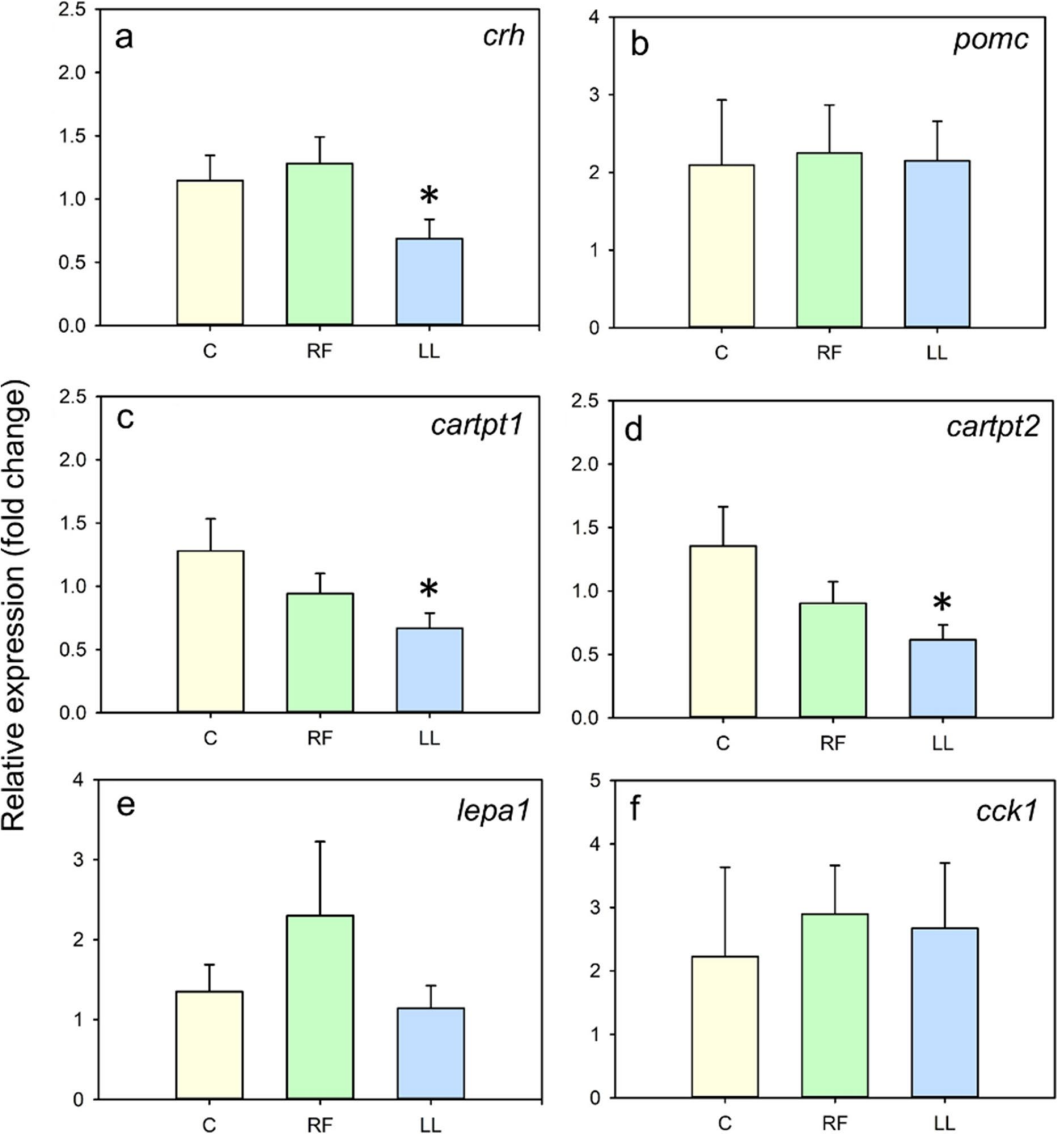
Effect of randomly-timed meals (RF) and continuous light (LL) in expression of anorexigenic factors in goldfish. Relative mRNA abundance of (**a**) *crh,* (**b**) *pomc,* (**c**) *cartpt1,* and (**d**) *cartpt2* in hypothalamus, (**e**) *lepa1* in liver, and (**f**) *cck1* in anterior intestine of goldfish. Data are expressed as mean + SEM. * *p* < 0.05 compared to control group, C (Student’s t-test used for all comparisons, except for C vs. RF comparison in panel e and for all comparisons in f, where Mann–Whitney U test was used)

The relative expression of orexigenic and growth-related factors is summarized in Fig. 6. RF fish showed upregulation (*p* < 0.05) of hypothalamic *hcrt (orexin)*, telencephalic *cnr1*(*cannabinoid receptor1*) and hepatic *igf1* (Fig. 6a,c,d). Conversely, LL fish showed decreased (*p* < 0.05) expression levels of orexigenic factors such as hypothalamic *hcrt* and *npy* (Fig. 6a,b), but higher expression of hepatic *igf1* (Fig. 6c), while *cnr1* expression remains unchanged (Fig. 6d). *Ghrelin* expression was also measured, but no changes were found (Online resource 1). Respecting the anorexigenic factors (Fig. 7), there were no significant differences in the expression of any of them in randomly-fed fish (Fig. 7c,d). LL fish showed a significant decrease in the expression of hypothalamic *crh*, *cartpt1* and *cartpt2* (Fig. 7a,c,d)*.* The amount of transcripts of *pomc*, hepatic *leptina1,* and intestinal *cck1* was similar in the three groups of fish (Fig. 7b,e,f).

## Discussion

The findings of this study provide insights into the effects of chronodisruption caused by either an irregular feeding schedule or continuous light exposure on the physiology and energy balance of goldfish. These conditions caused different alterations in the energy homeostasis, promoting a positive energy balance in randomly fed fish, but not in fish under continuous light.

### Effect of chronodisruption by randomly-timed meals on energy balance

The sustained absence of a regular fasting/feeding daily cycle had notable physiological effects in goldfish. Both the control and randomly-fed groups (both under 12L:12D photoperiod) showed higher levels of general locomotor activity during the light period and lower levels during the dark period, following the diurnal nature of this species (Iigo and Tabata 1996; Gómez-Boronat et al. 2018; Saiz et al. 2021). However, the RF group exhibited a slightly blunted day-night oscillation compared to controls. This is likely due to half of the meals being provided during the dark period in this group. This observation aligns with previous studies demonstrating that randomly fed gilthead seabream (*Sparus aurata*) exhibit higher locomotor activity levels (Sánchez et al. 2009). Regarding the feeding-associated locomotor activity, the RF group did not display FAA, in agreement with previous reports in fish fed irregularly (Sánchez et al. 2009; Saiz et al. 2021). The disappearance of FAA in RF fish occurred gradually over a few days after the start of the experiment.

Rhythmical patterns of clock genes and melatonin were not assessed in this study, as they have been extensively characterized in goldfish under different light and feeding conditions. Previous studies have consistently shown that the disruption of either the light/dark or the feeding/fasting cycle causes alterations in the rhythmicity of the molecular clock (Iigo and Aida 1995; Velarde et al. 2010; Nisembaum et al. 2012; Sánchez-Bretaño et al. 2015a, 2015b; Gómez-Boronat et al. 2018, 2022; Saiz et al. 2021; Alonso-Gómez et al. 2022; Navarro-Guillén and Yúfera 2025).

The metabolic rate remained higher in the RF group throughout the assay (around 4 h inside the metabolic chambers), suggesting that energy expenditure was higher in these fish. This result may be associated with higher stress levels, since it is known that stress significantly increases the metabolic rate in fish (Killen et al. 2021; Herrera-Castillo et al. 2024). A significant increase in cortisol levels has been previously reported in this species (Saiz et al 2021) and in gilthead seabream, also fed at random times (Sánchez et al. 2009). These results are consistent with previous data from rainbow trout (*Oncorhynchus mykiss*), where a chronic increase in circulating cortisol raised maintenance costs by mobilizing energy reserves, thus increasing the metabolic rate (Pfalzgraff et al. 2021). Beyond the role of cortisol, neurobehavioral alterations may also contribute to the heightened metabolic activity, since random feeding has shown to increase anxiety-like behavior in goldfish (Saiz et al. 2023). This could result from a constant state of vigilance for food, as recent research indicates that meal anticipation increases anxiety-like behavior in this species (Herrera-Castillo et al. 2025a).

Notably, feed intake was increased in randomly fed fish compared to controls, which could be explained by changes observed in some feeding-regulating transcripts causing an augmented appetite. Specifically, RF fish showed an upregulated expression of hypothalamic *hcrt*, and telencephalic cannabinoid receptor *cnr1,* which are known orexigenic signals in fish (Soengas et al. 2024; Volkoff 2024). Cannabinoids are involved in the hedonic control of feed intake in vertebrates, acting upon the reward system, and generating pleasurable sensations associated with positive reinforcement (Díaz-Rua et al. 2020; Bourdy and Befort 2023). These hedonic pathways may activate more strongly in fish that cannot predict the supply of food. As a result, food delivered unexpectedly might be perceived as more rewarding, leading to greater food consumption than under predictable feeding schedules. This heightened reward value associated with unpredictability is well established in behavioral science, where uncertain stimuli are generally considered more reinforcing than predictable ones (Robinson et al. 2023). Regarding anorexigenic neuropeptides, none of those analyzed at the hypothalamic, intestinal, or hepatic levels were significantly altered in the RF group. Considering that net feeding response results from the integration of multiple neuroendocrine signals, the lack of change in anorexigenic factors, combined with the upregulation of orexigenic signals, results in a positive orexigenic/anorexigenic balance, contributing to the increased feed intake observed under random feeding conditions.

It is important to consider that, in the present study, all metabolic parameters were assessed at a single time point to obtain a general overview of the metabolic and energetic status of the animals. This approach allowed us to detect robust differences between experimental groups, but it does not capture potential alterations in daily rhythmicity. In this regard, parameters such as plasma metabolites, cortisol, or feeding regulators are known to be rhythmic in fish, and their rhythms are affected by *Zeitgebers* like light and food (Delahunty et al. 1978; Tinoco et al. 2014; Saiz et al. 2021). Thus, chronodisruption may further affect the temporal organization of these variables beyond modifying their mean levels.

The increase in intake was also accompanied by higher body weight and enhanced growth. These results contrast with previous studies in teleosts, where gilthead seabream fed at random times showed equal or lower growth rates compared to controls (López-Olmeda et al. 2012). However, the present observations align with other studies conducted in mammals where mistimed meals have been associated with increased feed intake upon the first period in which food is offered, higher overall intake, and increased weight gain (Bray et al. 2013). Also in mammals, mistimed meals have been linked to metabolic syndrome and impaired glucose tolerance (Scheer et al. 2009). In line with this, plasma glucose levels were higher in RF goldfish, an effect that has also been documented in other randomly fed fish species such as gilthead seabream (López-Olmeda et al. 2012) and rainbow trout (Xu et al. 2022). The reduction in plasma triglyceride levels is consistent with previous findings in rainbow trout under random feeding conditions (Xu et al. 2022) and may indicate an altered lipid metabolism. However, this finding appears to conflict with the metabolic syndrome model, which typically presents hypertriglyceridemia. More detailed studies on lipid profile parameters and the activity of lipolytic and lipogenic liver enzymes are needed to clarify the underlying mechanisms.

Another remarkable finding in RF goldfish is their significantly larger liver size relative to body weight (hepatosomatic index). The liver serves as the primary organ for energy reserves in this species due to their very limited proportion of adipose tissue (Blanco et al. 2018). The hepatic concentrations of glycogen and triglycerides in RF fish were not significantly different from the controls, however, the increased proportional size of the liver in this group implies a higher net accumulation of hepatic glycogen and triglycerides relative to their body weight. Increased liver size because of chronodisruption has been previously reported in mammals, associated to alterations in lipid metabolism and liver fat accumulation (Sinturel et al. 2017; Borck et al. 2018). In the present study, the higher HSI could be attributed to a compensatory mechanism that allows fish to accumulate reserves in anticipation of possible food shortages when they are unable to predict when the next meal will be provided. The presence of such compensatory mechanisms following meal uncertainty has been suggested in other teleosts, as yellowfin seabream, *Acanthopagrus latus* (Tamadoni et al. 2020). This aligns as well with a broader evolutionary strategy observed across animals, where increased energy storage is used to mitigate the risk of starvation during periods of limited food availability (Anselme and Güntürkün, 2018).

### Effect of chronodisruption induced by continuous light on energy balance

In the group of goldfish under continuous light (LL), the general locomotor activity was uniform throughout the 24 h cycle, and the daily activity rhythm was only sustained by a peak following feeding time. These observations confirm that the chronodisruption protocol successfully disrupted the light entrainment of general locomotor activity. Regarding feeding-associated activity, both the LL and control groups displayed food-anticipatory activity (FAA). FAA increased progressively in the LL group, consistent with previous studies reporting that locomotor activity in goldfish becomes centered around feeding time when the light/dark cycle is removed and meals become a dominant cue (Feliciano et al. 2011; Saiz et al. 2021).

Fish under continuous light also displayed a dramatic increase in feed intake during the 8-h-long assay. Similarly, continuous light increases appetite in several fish species (Petit et al. 2003; Taylor et al. 2006; Volkoff et al. 2010; Türker and Yildirim 2011; Martínez-Chávez et al. 2021; Mondal et al. 2021), including goldfish (Konstantinov et al. 2002). The heightened feed intake observed in our study could be attributed to a shift in the balance of appetite-related genes, characterized by a marked downregulation of anorexigenic peptides such as hypothalamic *crh*, *cartpt1*, and *cartpt2*. However, despite this overall increase in feed intake, hypothalamic expression of other orexigenic neuropeptides (NPY and orexins) was reduced in LL goldfish. Similar results have been previously found in goldfish under LL conditions, with similar decreases in orexin expression, along with a loss of its rhythmicity, while NPY maintained a pre-feeding peak (Hoskins and Volkoff 2012). Altogether, these results suggest that while meal entrainment and FAA are still present, continuous light seems to differentially affect specific feeding-regulatory signals. The overall increase in intake caused by LL depends on the complex balance between all orexigenic and anorexigenic factors.

In support of this idea, the feeding-regulating signals affected by continuous light exposure in this work varied from those altered under random feeding conditions, suggesting that different neuroendocrine mechanisms may underlie the increased feed intake observed in these two chronodisruption models. This result corroborates the general acceptance that disruption of the light/dark cycle is likely to strongly affect light-entrainable oscillators, while disruption of the feeding/fasting cycle potentially impacts food-entrainable oscillators (Challet 2019). Despite these differences, circadian disruption in both conditions ultimately led to dysregulation of the feeding control systems.

Despite the LL group's inclination to consume higher amounts of food, similar to that observed in the RF group, the biometric parameters of the fish under continuous light resembled those of the controls, showing no significant differences in length, weight gain, SGR, or HSI. Conversely, most studies in other fish species that have lengthened the daily photophase or even removed the scotophase have observed increased growth (Boeuf and Le Bail 1999; Martínez-Chávez et al. 2021; Thraya et al. 2025). Such increase in growth could be due to higher feed intake and has sometimes been linked to an improved feed conversion efficiency (Boeuf and Le Bail 1999; Martínez-Chávez et al. 2021). However, the current evidence seems to indicate that this is not the case for goldfish. The present findings, along with a previous study showing that continuous light decreased growth rate and feed utilization in this species (Konstantinov et al. 2002), suggest that the growth-promoting effects of continuous light do not extend to *C. auratus*. A similar pattern has been observed in common carp (*Cyprinus carpio*), a cyprinid species closely related to goldfish, where 60 days of continuous light exposure significantly reduced both the final body weight and the specific growth rate compared to 12L:12D (Ghomi et al. 2011). No increase in growth was also reported in *Pleuronectes ferrugineus* or in *Astronotus ocellatus* under LL (Purchase et al. 2000; Zare et al. 2024). These differences may reflect species-specific sensitivities to photoperiod manipulation, or alternatively, may depend on the duration and context of LL exposure, and the feeding protocols.

Moreover, it is important to note that continuous light has been recognized as a potential stressor for fish, and the most negative impacts become evident after prolonged exposure periods (Malinovskyi et al. 2022). For instance, in largemouth bass (*Micropterus salmoides*) constant light exposure improved weight gain during the first 4 weeks, but negative effects were noticed after 16 weeks, with a significant reduction in body weight, SGR, and HSI (Malinovskyi et al. 2022). In fact, although average cortisol levels in the present study showed a tendency to increase under LL conditions—without reaching statistical significance—other evidence points to the activation of a stress response. For instance, in goldfish exposed to similar continuous light conditions, anxiety-like behaviors were significantly increased, and the individual variation in cortisol levels between the beginning and end of the experiment was also greater compared to control conditions (Saiz et al. 2023). Likewise, in *A. ocellatus*, continuous light worsened the stress response during acute confinement (Zare et al. 2024). These findings suggest that continuous light exposure can affect both behavioral and endocrine responses, raising concerns for animal welfare and questioning the overall benefits of continuous light in aquaculture practices.

The fact that no increased growth was observed in LL fish despite their higher appetite and elevated *igf1* levels suggests a decoupling between growth-related endocrine signals and somatic growth. This mismatch may be explained by an increased energy expenditure under continuous light conditions. Supporting this notion, LL fish also showed an increased rate of oxygen consumption, as has been reported in several fish under artificial light at night (ALAN) or continuous light (Imsland et al. 1995; Pulgar et al. 2019; Wei et al. 2019), including goldfish (Konstantinov et al. 1999). Considering that under a standard photoperiod, the metabolic rate in goldfish is rhythmic with higher values during the light phase (Herrera-Castillo et al. 2024), it is likely that the metabolic rate rhythm in the LL group was disrupted, preventing the metabolic rate from falling during the subjective night and increasing overall energy use. This may contribute to the greater energy expenditure observed in LL fish. A similar situation can be hypothesized about the plasma glucocorticoid rhythm. Even if the cortisol levels during the daytime were only slightly higher than in controls, their daily oscillation may be blunted in LL fish, lacking the usual decrease during the inactive phase, as reported previously in animals exposed to ALAN (Abílio et al. 1999; Van Der Meer et al. 2004; Fonken and Nelson 2014). Further studies focusing on measuring energy expenditure indicators at multiple time points throughout the 24 h would help clarify these questions. Another possible contributor to increased energy expenditure could be a more intense swimming activity, according to several studies where continuous light or lengthened photophase lead to hypermobility in animals (Singh et al. 2013; Navarro et al. 2014; Pulgar et al. 2019). Furthermore, goldfish under continuous light show an increase in swimming velocity in the open field test compared to control and RF groups (Saiz et al. 2023). Thus, according to our study, the exposure to continuous light in goldfish appears to promote energy loss rather than gain, and therefore could be detrimental for farming. To sum up, these results support the relevance of carefully managing light exposure, such as implementing appropriate photoperiods and minimizing large disruptions in the light/dark cycle, to promote greater welfare of fish.

## Conclusions

Overall, our results show that disruption of light and feeding cues alters the circadian system, leading to neuroendocrine and metabolic changes. Both chronodisruption protocols caused dysregulation of feeding control systems, inducing hyperphagia, but through different mechanisms. Disturbance of food-entrainable oscillators increases body weight and hepatic energy storage, resulting in a positive energy balance despite an elevated metabolic rate. On the other hand, fish exposed to continuous light had no changes in weight or growth, probably due to an increased energy expenditure. Thus, these findings emphasize the need for careful management of feeding schedules and light conditions in aquaculture settings to optimize the welfare, growth, and overall performance of farmed fish.

## Supplementary Information

Below is the link to the electronic supplementary material.

Supplementary file1 (PDF 193 KB)

## Data Availability

The datasets generated during and/or analysed during the present study are available in the UCM database DOCTA (https://hdl.handle.net/20.500.14352/134899).
