# Supplementary material for: Feeding and light cycle disruptions have distinct effects on energy balance and neuroendocrine regulators in goldfish
Source: Fish Physiol Biochem. 2026 Apr 24;52(3):66. doi: 10.1007/s10695-026-01685-1 (PMC13109174; doi:10.1007/s10695-026-01685-1)
Supplement: Supplementary file 1 — Supplementary file1 (PDF 193 KB) [file 10695_2026_1685_MOESM1_ESM.pdf]

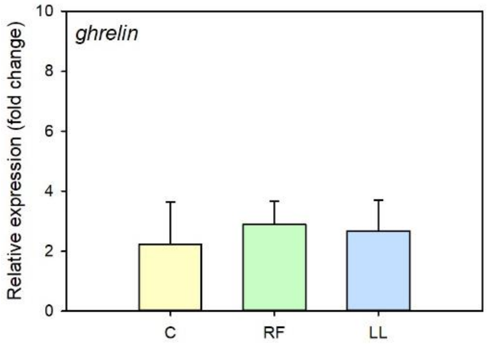

Online resource 1: Effect of continuous randomly-timed meals (RF) and continuous light (LL) in expression of ghrelin in the anterior intestine of goldfish. Relative mRNA abundance. Data are expressed as mean + SEM. n=6-10/group.
